# General Practitioners’ Experiences of, and Responses to, Uncertainty in Prostate Cancer Screening: Insights from a Qualitative Study

**DOI:** 10.1371/journal.pone.0153299

**Published:** 2016-04-21

**Authors:** Kristen Pickles, Stacy M. Carter, Lucie Rychetnik, Kirsten McCaffery, Vikki A. Entwistle

**Affiliations:** 1 Centre for Values, Ethics & the Law in Medicine, University of Sydney, Australia; 2 School of Medicine, University of Notre Dame, Sydney, Australia; 3 School of Public Health, University of Sydney, Australia; 4 Health Services Research Unit, University of Aberdeen, Scotland; Carolina Urologic Research Center, UNITED STATES

## Abstract

**Background:**

Prostate-specific antigen (PSA) testing for prostate cancer is controversial. There are unresolved tensions and disagreements amongst experts, and clinical guidelines conflict. This both reflects and generates significant uncertainty about the appropriateness of screening. Little is known about general practitioners’ (GPs’) perspectives and experiences in relation to PSA testing of asymptomatic men. In this paper we asked the following questions: (1) What are the primary sources of uncertainty as described by GPs in the context of PSA testing? (2) How do GPs experience and respond to different sources of uncertainty?

**Methods:**

This was a qualitative study that explored general practitioners’ current approaches to, and reasoning about, PSA testing of asymptomatic men. We draw on accounts generated from interviews with 69 general practitioners located in Australia (n = 40) and the United Kingdom (n = 29). The interviews were conducted in 2013–2014. Data were analysed using grounded theory methods. Uncertainty in PSA testing was identified as a core issue.

**Findings:**

Australian GPs reported experiencing substantially more uncertainty than UK GPs. This seemed partly explainable by notable differences in conditions of practice between the two countries. Using Han et al’s taxonomy of uncertainty as an initial framework, we first outline the different sources of uncertainty GPs (mostly Australian) described encountering in relation to prostate cancer screening and what the uncertainty was about. We then suggest an extension to Han et al’s taxonomy based on our analysis of data relating to the varied ways that GPs manage uncertainties in the context of PSA testing. We outline three broad strategies: (1) taking charge of uncertainty; (2) engaging others in managing uncertainty; and (3) transferring the responsibility for reducing or managing some uncertainties to other parties.

**Conclusion:**

Our analysis suggests some GPs experienced uncertainties associated with ambiguous guidance and the complexities of their situation as professionals with responsibilities to patients as considerably burdensome. This raises important questions about responsibility for uncertainty. In Australia in particular they feel insufficiently supported by the health care system to practice in ways that are recognisably consistent with ‘evidence based’ professional standards and appropriate for patients. More work is needed to clarify under what circumstances and how uncertainty should be communicated. Closer attention to different types and aspects of the uncertainty construct could be useful.

## Introduction

Prostate-specific antigen (PSA) testing for prostate cancer is controversial. There are unresolved tensions and disagreements amongst experts, and clinical guidelines conflict. This both reflects and generates significant uncertainty about the appropriateness of testing, especially in asymptomatic men. The United States Preventive Services Taskforce (USPSTF) recommends physicians should not offer or order PSA screening unless they are prepared to engage in shared decision making (SDM) that enables an informed choice by the patient; this includes providing information about the associated uncertainties [1]. The Royal Australian College of General Practitioners (RACGP) advises GPs not to raise the issue of PSA testing unless men specifically ask, in which case they should provide full information regarding the benefits, risks, *and uncertainties* (about benefits and risks) [2]. The UK’s National Screening Committee (UK NSC) policy similarly does not recommend universal screening for prostate cancer. Instead there is an informed choice program in place where men who request PSA testing can have it following detailed information exchange to aid shared decision making.

Primary care clinicians advise on and are gatekeepers to the PSA test. In practice, they vary in what they disclose to patients about the uncertainty and controversy that surrounds it. Recognition of uncertainty may in general be ethically preferable, facilitating more completely-informed consent [3] and promoting realistic patient expectations about medical care [4,5]. However, research from the US and UK suggests communication of uncertainties with patients in the context of PSA testing is infrequent and complex [6–8].

Uncertainty is a common but under-researched issue in general practice and clinical decision making [9,10]. Some research about communication in various clinical settings indicates that doctors can be reluctant to disclose uncertainty, preferring to present the appearance of certainty to their patients [11,12], and to avoid being judged as inadequate or ineffective [13]. There are different findings (and suggestions about the implications of) communicating uncertainty. Communicating uncertainty can have a negative effect on patients, including heightening perception of risk, causing unnecessary worry [14], and decreasing ability to make decisions about care [15]. In contrast, other research suggests honest expressions of uncertainty may improve the doctor patient relationship [13], facilitating trust [11], therapeutic effectiveness [16] and patient confidence [10], and decreasing patient interest and participation in medical screening [6,17,18].

Studies investigating doctors’ experiences of uncertainty, specifically in the context of PSA testing, are scarce. The experience of uncertainty is a challenging phenomenon to explore [14]; yet it is central to much of medical practice. Some argue that tolerance of uncertainty is an essential dimension of professional competence [19]. Others have suggested that changing professional and public attitudes towards medical error and uncertainty is key to reducing overdiagnosis and overtreatment [20].

Han’s taxonomy of uncertainty [14] makes a valuable contribution to its conceptualisation in health care. As shown in Box 1 (modified), the taxonomy has three dimensions: sources of uncertainty (where uncertainty comes from), issues of uncertainty (what uncertainty is about), and locus of uncertainty (who is uncertain).

**Box 1. Han’s (2011) taxonomy of uncertainty: a summary.**

Sources of uncertainty:

1. *Probabilistic* uncertainty generated from the indeterminacy of a phenomenon’s future outcome, such as the probability of benefit (or harm) from a test or treatment
2. *Ambiguity* signifies the lack of reliability, credibility, or adequacy of information about a phenomenon of interest, and includes imprecision (e.g. wide probability estimates of benefit /harm from treatment), conflicting opinions/evidence, and lack of information
3. *Complexity* is uncertainty arising from aspects of a phenomenon itself, which make it difficult to comprehend; e.g. numerous potential outcomes from a medical test or treatment or the existence of varied risk factors, symptoms, or signs of a given disease.

Issues of uncertainty:

1. *Scientific* uncertainty is disease-centred. Encompasses uncertainties about diagnosis, prognosis, causal explanations, treatment recommendations
2. *Practical* uncertainty is system-centred. Applies to the structures and processes of care (competence, quality, responsibilities)
3. *Personal* uncertainty is patient-centred. Psychosocial and existential issues (relationships, impact on life goals)

Locus of uncertainty:

Where the uncertainty resides: with the clinician or the patient

In this paper we first report on clinician perspectives and experiences of uncertainty in relation to PSA testing using Han’s framework. We then add to Han’s taxonomy an outline of the strategies that GPs use to manage uncertainty in PSA testing.

We use data from a qualitative study that explored general practitioners’ current approaches to, and reasoning about, PSA testing of asymptomatic men. Uncertainty in PSA testing was identified as a core issue, and we draw on this data to address the following questions:

1. What are the primary sources of uncertainty as described by GPs in the context of PSA testing?
2. How do GPs experience and respond to different sources of uncertainty?

## Methods

### Design

We applied the well-established, systematic qualitative research methodology, grounded theory [21]. All study procedures were approved by the Cancer Institute NSW and the University of Sydney Human Research Ethics Committee [#15245]. GPs had an opportunity to discuss the study, and gave written consent, prior to participation.

### Participants and Setting

We recruited a sample of 69 GPs (40 Australian, 29 UK) for this study. In Australia we advertised via the newsletters and email lists of GP organisations (Medicare Locals) in Sydney, in mass and social media, and in medical journals. Rural GPs were accessed by phoning practice managers, through colleagues, and advertising with rural Medicare Locals [22].

We included GPs from the United Kingdom to also explore PSA testing reasoning and practice in a jurisdiction with comparatively lower rates of prostate cancer screening than Australia. We subsequently recruited 29 GPs throughout England (n = 24) and Scotland (n = 5). Our initial sample of GPs responded to an invitation distributed by academic colleagues through professional networks. We then broadened our sample by advertising via email to members of the Royal College of General Practitioners (RCGP), primary health care departments, university academic departments, and general practice and research mail lists. We also advertised via newsletter including the Society for Academic Primary Care (SAPC) and RCGP Scotland’s eBulletin.

GPs were invited to contact KP if they were interested and willing to participate. Participating GPs were of varying ages, clinical experience, gender, and patient populations. All GPs who expressed interest in participating were included. GPs were compensated financially for their time.

### Interviews / Data Collection

We generated data via in-depth interviews. The semi-structured interview schedule covered a broad range of topics, including GPs’ recent clinical encounters involving PSA testing decisions; communicating information; screening pathways; and overdiagnosis. The schedule was modified between interviews, informed by the developing analysis. Uncertainty was not specifically included as a topic for discussion in the schedule; rather it was a recurring concept that was identified during data analysis. Interviews with Australian GPs took place between March 2013 and June 2014 and with UK GPs between September and December 2014. They were all conducted by KP, mostly by telephone and Skype, and ranged in duration from 18 to 70 minutes. All interviews were audio-recorded and transcribed verbatim.

### Data Coding and Analysis

The analysis was led by KP, who coded the transcripts and wrote detailed memos which were regularly reviewed and discussed by the authors in analysis meetings. A subset of transcripts was read and coded by three authors independently; this coding was compared and discussed to inform the development of the central concepts in the study.

A longstanding point of contention in grounded theory methodology is the relation between the theory being produced, which is ‘grounded’ in the data collected, and existing relevant theory. While early expressions of grounded theory methodology [23] strongly emphasised the development of new theory *as opposed* to the testing of existing theory, contemporary mainstream grounded theorists strongly concur that qualitative empirical work must be conducted in the context of existing knowledge [24]. As uncertainty was identified as a core category in our data analysis, we turned to the literature to develop a better understanding of the concept, and identified Han’s taxonomy of uncertainty in health care [14]. This taxonomy resonated with our interpretation of the data and suggested face validity for our early analysis of the sources and issues of uncertainty. We used Han’s taxonomy to develop our analysis of GPs’ experiences with uncertainty and categorised our data according to the ‘sources’ and ‘issues’ of uncertainty as described in the framework. In addition, we developed a new set of concepts related to how GPs respond to uncertainty in PSA testing, an issue that was not included in Han’s typology.

## Results

We identified considerable variation in GPs’ interpretation, management, and experiences of uncertainty in terms of the source of the uncertainty they described, its impact on usual practice, and GPs perception of who should respond to uncertainty.

There seemed to be substantially more uncertainty experienced among Australian than UK GPs, perhaps partly explainable by the notable differences in conditions of practice for PSA testing between the two countries. The United Kingdom system is structured in several ways likely to decrease uncertainty. There is a clear policy directive against screening asymptomatic men for prostate cancer. There is an established norm of communicating with men who ask about PSA testing, and a structured approach to communication including a written information resource. In addition, referral pathways following particular test results are well-defined. In contrast, Australian policy is not clearly defined or directive, and at the time of this study there was no single authoritative document advising GPs how or what to communicate to men. The lack of policy clarity seems likely to contribute to the considerable variation in GP approaches to PSA testing [22].

Unsurprisingly, given these differences, Australian and UK GPs talked differently about prostate cancer screening. Asymptomatic men ask about prostate cancer screening frequently in Australian practice. Yet Australian GPs said they felt unsure about what is the “right” thing to do about PSA testing, expressed frustration about the lack of formal guidance to direct their practice, and many found talking with men about PSA testing a challenging experience because of underlying uncertainty. In contrast, the majority of GPs practicing in the UK were not routinely having discussions with asymptomatic men about PSA testing. They explained that screening men for prostate cancer is not a widely supported process, nor a common request from patients. When men did want a PSA test, GPs favoured providing them a standard government-produced information leaflet, to promote informed decisions. UK GPs considered conversations about PSA testing with asymptomatic men to be of low priority unless men asked, and overall did not express any notable uncertainty about whether to test men or not. As a result, there was comparatively less UK data about UK GP experiences of uncertainty. The results described below therefore predominantly describe the Australian data. We will return to the implications of this in the discussion.

### Where Does GPs’ Uncertainty Come from and What Is the Uncertainty About?

Table 1 outlines sources of GPs’ uncertainty about PSA testing. We have included Han’s definition of each source of uncertainty followed by a summary of how GPs described this type of uncertainty manifesting in their practice.

**Table 1:** Sources of uncertainty (where is GP uncertainty coming from?).

Han’s SOURCES of uncertainty	How does this taxonomy manifest in the context of PSA testing?
**PROBABILISTIC UNCERTAINTY** Generated from the indeterminacy of a phenomenon’s future outcome, such as the probability of benefit (or harm) from a test or treatment	Several important potential outcomes may follow from PSA testing. Early diagnosis and treatment may decrease prostate cancer death for a small number of men. For the majority, any mortality benefit is outweighed by risk of harm: testing and treatment is associated with substantial harms, including impotence, incontinence, and anxiety. Although the probabilities of some of these outcomes can be estimated for populations, there is no way of knowing which individual patient will experience which outcomes.
**AMBIGUITY** Lack of reliability, credibility, or adequacy of information about a phenomenon of interest; includes imprecision (e.g. wide probability estimates of benefit /harm from treatment), conflicting opinions/evidence, and lack of information	The PSA test performs poorly as a screening tool. It is known that some screen-detected prostate cancers are more aggressive than others, but the PSA test cannot differentiate aggressive from non-aggressive cancers. This, together with uncertainties about treatment effects, uncertainty about how particular patients might react to different biomedical clinical outcomes (physically and psycho-socially), and how patients may respond differently to the risk of these outcomes, means it is unclear what test results might actually mean for each individual patient both at the point of testing and following an abnormal test result
**COMPLEXITY** Arising from aspects of a phenomenon itself, which make it difficult to comprehend; e.g. numerous potential outcomes from a medical test or treatment or the existence of varied risk factors, symptoms, or signs of a given disease. Confounding, interacting factors add complexity and complicate interpretations and outcomes. Personal judgment and clinical experience informs decisions.	The multiple-stage, multiple possibility sequence of testing and treatment outlined above add complexity to an evaluation of testing. Although the patient descriptors used in research studies and guidelines may seem simple (e.g. age 70+, asymptomatic) in general practice many individual patients are complex in ways not reflected in the evidence base. This includes the presence of comorbidities, and the difficulty in distinguishing symptomatic from asymptomatic men because of the multiplicity of causes of the symptoms commonly associated with prostate cancer. GPs consequently feel uncertain about how clinical descriptors should be applied.

Table 2 captures the issues of uncertainty, again presented with Han’s definition of the issues followed by specific examples in each cell from our data. Han’s framework characterises ‘personal uncertainty’ as patient-centred. However because this study focused on the perspective of GPs, our data also includes personal uncertainty with a locus in GPs.

**Table 2:** Issues of uncertainty (what is the uncertainty ABOUT?).

Han’s ISSUES of uncertainty (what is the uncertainty about?)	Probabilistic material as a Source of uncertainty (uncertainty arising from the probabilistic nature of information)	Ambiguity as a Source of uncertainty (uncertainty arising from the ambiguity of evidence or expert guidance):	Complexity as a Source of uncertainty (uncertainty arising from the interaction of multiple factors, some unknown):
**SCIENTIFIC UNCERTAINTY** *Disease-centred*. Uncertainties about diagnosis, prognosis, causal explanations, treatment recommendations	• GPs concerned about their inability to predict clinical outcomes (such as incontinence or impotence) following testing and treatment at the individual patient level. Probabilities can predict aggregate outcomes in a population, but cannot specify their exact distribution, or the probable severity of potential outcomes in any given individual.	• GPs concerned about conflicting estimates for particular outcomes for particular populations GPs uncertain about the conclusions that should be drawn from the evidence base for/against screening for prostate cancer	• Interpretation of the benefits and risks of testing and treatment can change over time and depend on various assumptions (e.g. about patient values and current state). GPs concerned about evaluating benefits and risks and making treatment decisions relating to individual patients because of this complexity.
**PRACTICAL UNCERTAINTY** *System-centred*. Uncertainties about the structures and processes of care (competence, quality, responsibilities)	• GPs described probabilities as challenging to think about and apply in individual clinical encountersGPs unsure how urologists will work with patients referred with a high PSA result	• GPs uncertain about professional practice due to disagreement between guidelines GPs concerned about conflicting guidance from medical authority: professional organizations and colleagues vary in the recommendations they make about whether or not (and under what circumstances) to screen with PSAGPs unclear under what conditions they could be medically liableGPs concerned about inconsistent referral pathways and advice	GPs find communicating probabilistic information with specific patient types (e.g. health illiterate, anxious, determined to have the test) difficultGPs seeing a patient who usually consults a different GP found this a complex and ‘awkward’ situation in which to practice if their testing preferences were dissimilar to the GP they were replacing
**PERSONAL UNCERTAINTY** *GP/patient-centred*. Uncertainties about psychosocial and existential issues (relationships, impact on life goals)	GPs concerned about their inability to predict the psychological and existential outcomes of testing and treatment that would be experienced by the patient	• GPs consider what is at stake for them as an individual clinician—legally, psychologically, professionally and socially—if they do or do not testGPs uncertain about what is the right thing to do in this context in order to be considered a ‘good GP’ and preserve relations with their patientsGPs concerned about whether it is ok to change PSA testing practice following personal and practice experiences	• GPs concerned about their ability to judge how ‘good’ any individual patient’s consent/decision might be, and what the outcomes of poor/inaccurate judgment may mean for them and their patientsGPs uncertain about individual patient tolerability of potential consequences of their decisionsGPs feel conflicted when their own personal preferences for testing/not testing conflict with advice they provide

Our data indicate that GPs may experience a diverse range of uncertainties with respect to PSA testing. There were important differences however, between Australian and UK GPs’ descriptions regarding what their uncertainty was about. Australian GPs’ uncertainty was related to all three of the issues described above: scientific, practical, and personal. UK GP experiences of uncertainty were mostly about personal issues, because (a) GPs were clear about procedural expectations coming from government and medical bodies about PSA testing, and scientific uncertainty was dealt with via clear guidelines and norms; and (b) UK GPs expressed a sense that the medical profession was collectively managing the uncertainty so individual GPs were facing less practical uncertainty because the UK system has processes in place to help them manage it. Thus the UK GPs’ uncertainty was predominantly patient-centred; they were mostly concerned that when their patients sought or asked about PSA testing, they were then burdened with uncertainty due to the uncertain nature and quality of the available information. UK GPs did not feel uncertain themselves but worried that their *‘patients have to make well-informed decisions and I suppose that’s where it’s such a minefield of uncertainty*, *it must be very difficult for people to say that they’ve made a well-informed decision’* (UKGP26).

Below we outline strategies GPs described using to handle uncertainty in the context of PSA testing of asymptomatic men. The information below draws primarily from the Australian data; where observations are based on UK data this is noted.

### What Strategies Do GPs Use to Manage Uncertainty?

We identified three main approaches GPs used when faced with uncertainty around PSA testing, specifically about decision-making and responsibility:

1. Sometimes GPs *took charge* of uncertainty, considered it a usual feature of their daily practice, and managed (at least some) uncertainties on their own
2. Sometimes GPs *engaged others* in managing uncertainty: they accepted some uncertainty as a challenge and used it as an opportunity to engage the medical profession, colleagues, and patients about how the uncertainty would be handled, to enable them to better support patients and find shared solutions
3. Sometimes GPs *sought to transfer* to other parties the responsibility for reducing or managing some uncertainties.

Some GPs tended to manage all types of uncertainty about PSA testing in a relatively consistent way, adopting one of the three approaches described above (i.e. some GPs usually took charge, usually accepted and engaged, or usually transferred, although this was never absolute). Other GPs were more variable in their approach, applying different management strategies in different situations. Particular sources of uncertainty also tended to elicit particular types of responses. So GPs who tended to use different strategies to manage different types of uncertainties may call upon one or all of the techniques depending on the type of uncertainty, the particular situation, and the GPs individual interpretation of it and level of tolerance.

We describe the three categories of GPs approaches below.

#### 1. GPs taking charge of uncertainty

When we describe GPs as “taking charge of uncertainty” we refer to circumstances when they recognised uncertainty, tolerated it, and accepted it as a lasting presence in their practice. They found ways through the uncertainty for themselves according to their *‘own protocol’* (GP24) because *‘the scientists and the doctors cannot tell you what’s going to happen’* (GP17). GPs taking charge had settled into ways of dealing with some kinds of uncertainty and now just got on with it, acting confidently as lead decision-makers. It depended on the individual GP whether “taking charge” occasioned active recommendation of PSA testing or not.

Although these GPs might seek more medical information to inform further decisions following initial testing (e.g. actively PSA testing including repeat testing, ordering alternative tests), this was independent of external parties: they did not describe feeling any pressure to consult patients or recommendations before making decisions. Some GPs preferred to practice using a *‘gut feeling sort of approach’* (GP21), because *‘nobody really knows the right answers to any of these questions’* (GP18). For them, *‘because the science is imperfect*, *then personality and medical judgement have…more of an important role’* (GP17). These GPs directed their PSA testing practice according to their clinical experience.

When GPs took charge of uncertainty about testing decisions, they did not routinely discuss uncertainty comprehensively with patients. Sometimes they believed a patient did not have the ability to cope with the complex information, and sometimes they assumed a position of making decisions on behalf of patients to protect them from navigating uncertain decisions, *‘I think it’s a lot easier for the patient to not be in that position [of making decisions grounded in uncertainty] at all’* (GP17). There were some GPs who disagreed with this as an appropriate approach; however the GPs using it as a strategy did so to protect their patients from the uncertainty in current knowledge.

When GPs took charge, they did not perceive or experience uncertainty as burdensome: they simply accepted that *‘it’s not clear and that’s just the way it is’* (GP20), or ‘[the PSA test is] *not perfect but it’s all we’ve got’* (GP26). One GP commented that all doctors should have the capacity to make decisions about the evidence around PSA testing, despite its uncertainty *‘if you think it’s too hot in the kitchen*, *get out…I have no sleepless nights worrying about missing one*, *I think that’s just–just life’* (GP18). Ultimately, these GPs were comfortable acknowledging that GPs *‘don’t have to have the answers to everything in medicine’* (GP3).

Interestingly, how to handle *normal* PSA test results was a source of uncertainty for some UK GPs because all guidance following PSA testing is for symptomatic patients or abnormal test results. UK GPs really relied on having guidelines to direct their practice. These guidelines are, roughly: 1) don’t test; 2) if someone happens to ask, give them this information; 3) if abnormal PSA result, refer to this hospital (clear procedure). As a result GPs said they were uncertain about how to proceed following normal PSAs. Should they, for example, tell the man that they could now stop worrying altogether, or that they should come back in x years? They wanted to avoid being in this position of uncertainty so their strategy was to, wherever possible, not test in the first place. In comparison, some Australian GPs described normal results as a source of relief and reassurance for them and their patients, because for them, the uncertainty of not knowing was greater than knowing a test result (abnormal of not). GPs told of how most patients in Australia receiving PSA testing expect to be tested annually, so GPs actively test them year-to-year in the hope of finding another normal result.

#### 2. GPs engaging others in managing uncertainty

In some situations, GPs were committed to engaging others in managing uncertainty about PSA testing. They took uncertainty as a challenge, and engaged the medical profession, colleagues, and patients in their quest to make a good decision in the context of shared uncertainty. Engaging others occurred via relationships and communication. These GPs took it as their duty to grapple with the uncertainty and felt a sense of responsibility to share it with the profession (usually as a member of an organisation of GPs and specialists), and with patients: to inform them of uncertainty, ongoing debate and lack of consensus. They expected to be supported by GP and specialist colleagues to help manage their own emotional and informational needs (e.g. consulting colleagues and specialists for advice), and to in turn effectively support patients to make decisions.

Some GPs negotiating uncertainty felt more comfortable than others managing the uncertainty of the ‘grey zone’ of PSA test results. In this zone (>4ng/ml, <10ng/ml) the clinical implications of test results, and decisions about what to do, are most contested. GPs comfortable with managing the grey zone themselves via repeat testing or active surveillance (rather than external referral to a specialist) had *‘no hesitation’* (GP31) to contact colleagues or specialists for advice and *‘reassurance…of what to tell the patient’* (GP5) once uncertainty moved beyond the GPs’ *‘own level of comfort and expertise’* (GP31).

GPs who negotiated uncertainty were more inclined to talk about the uncertainty of PSA testing with their patients. They reported telling their patients that it is not possible to be sure about aspects of PSA testing, including probabilistic outcomes and individual prognosis, and therefore any advice offered had some degree of underlying uncertainty. GPs told patients they themselves don’t know what to do about PSA testing of asymptomatic men and don’t *‘pretend to fully understand it’* (GP31), *‘so I don’t expect patients to have the capacity to–we say fully informed*, *but really we’re not*, *so how can the patient be*?*’* (GP26). Yet these GPs were adamant that regardless of the limitations of the available knowledge base, *‘the information needs to be on the table’* (GP31).

GPs who talked to patients about the uncertainties of PSA testing said they acknowledged the discord in professional opinion about what should be done with their patients. For many GPs this was a source of considerable frustration: *‘every week and I’m like*, *for God’s sake*, *can someone make a decision so I know what to do*?*’* (GP26). They often did not feel supported by the medical profession: *‘It’s up to individual GPs to sort it out himself–I mean it shouldn’t happen this way*, *but we’re not getting really helpful information from our so-called experts’* (GP28). The RACGP guidelines (as outlined in the introduction) were described as unhelpful, *‘a blanket ethical statement’* (GP28) and GPs said they felt *‘we’re still in a mess with what we’re actually advising men to do…we don’t know what the hell we’re doing’* (GP8). Some of these GPs reported clear ideas about how authorities should respond to support GPs and to support patients. This centred on consensus, talking the same language, and telling GPs exactly what information they should provide patients. Some GPs did, however, recognise the difficult position authorities are in when trying to produce policy in a complex situation; *‘the opinions and the spectrum…it just reflects that it’s unclear and that opinion is divided depending on who you talk to’* (GP20).

For many GPs an element of discomfort with uncertainty was ever-present. GPs felt guilty about possible negative psychosocial effects on their patients of understanding how messy and complex the situation is; ‘*the spiel that I give men about this leaves them with virtually no ability to make a good decision…I don’t make things easier for people’* (GP8). GP attempts to share their knowledge of the uncertainties and experience of being uncertain about what to do with patients sometimes proved futile: for example if the patient wanted the GP to decide what to do on their behalf and preferred not to be engaged in/take on the lead role in decision making.

Of the three approaches, it was the GPs engaging others in the uncertainty of PSA testing who were most likely to experience that uncertainty as burdensome. They worked hard to make an impossible situation as good as possible. But their work to mitigate the uncertainty was unrecognised and unrewarding; the more GPs tried to wrestle with the uncertainty, the more uncertain the situation appeared. Yet these GPs continued to take on some uncertainty as a challenge because doing something–engaging patients and the profession–felt appropriate and ‘right’ to them as GPs.

#### 3. GPs transferring uncertainty

Finally, some GPs employed strategies of “transferring” responsibility for decision making in the face of uncertainty. They perceived uncertainty as problematic and uncomfortable and sought out other parties to reduce their experience of uncertainty. The external authorities could include: urologists, those researching the test, legal authority, or the health system. One GP described this process of transferring responsibility for decision-making as *‘handballing it to somebody else’* (GP26).

In practice, GPs in this category were not likely to repeat PSA test results that returned in the grey zone, rather preferring to refer those patients immediately to specialists to make decisions about the next stage of management. In fact a subset of GPs were committed to immediate referral regardless of what the PSA test result was: ‘*even though it’s [PSA] still well within the normal range for their age—I just think that’s a specialist’s [urology] decision*, *not mine’* (GP26). For GPs, having urology as a backup meant they could regard uncertain PSA results as *‘not my problem*, *quite frankly’* (GP25).

Some GPs engaged in active PSA testing as a strategy for managing their uncertainty, particularly about potential legal ramifications of not testing; *‘I would probably still send him for the test because I’d be worried somebody would sue me if I didn’t*’ (GP22). One GP said that when in a position of not knowing what to do *‘I think medico-legal comes into that…you’re more defensive in your acting’* (GP34). This subgroup of GPs looked to legal authority to protect them and justify their practice, and perceived medico-legal obligations influenced their practice particularly when they felt uncertain about what to do.

Some GPs left decision making entirely up to the individual patient to deal with; ‘so I say well it’s your decision and if you want to have it, you can. If you don’t want to worry about it, that’s ok with me too. So I let them decide basically’ (GP21). A number of practices in Australia had implemented their own policy: a recall system whereby patients would be automatically notified when due for a PSA test with an accompanying pathology form. In these instances, GPs minimised repeated engagement with the uncertainties of PSA testing and personal responsibility for patient decision-making. By automating the process they effectively transferred responsibility for follow-up to a practice management system, and their patients would in turn choose how to respond to the automated invite.

Those GPs whose default-practice was to transfer responsibility had worked out a way of practicing which relieved them of the burden of advising and making practice decisions based on uncertain foundations (at least until their next consultation involving a PSA test). This risk-averse approach–transferring uncertainty to external authority as soon as possible–was perceived by GPs as a way to save patients from being burdened by GP uncertainty and meant the GP did not have to engage with what they considered an unresolvable situation.

### How Do GPs Respond to Different Sources of Uncertainty?

Probabilistic uncertainty was identified as a major source of uncertainty by the GPs, yet they tended to speak of it as being more tolerable than uncertainties arising from ambiguity and complexity. Overall, GPs accepted responsibility for probabilistic uncertainty and shared their knowledge about it; it was not as common for GPs to attempt to transfer responsibility for this source of uncertainty. Many GPs described handling probabilistic uncertainty reasonably comfortably on their own. They saw it as central to the GP role and to clinical judgment, which necessarily involves interpreting scientific evidence and probabilities. Individuals or organisations were not blamed or held directly responsible for probabilistic uncertainty. GPs described probabilistic uncertainty as challenging (e.g. indeterminate medical outcomes), but had mostly found a relatively settled way of dealing with it because they had few alternatives.

The two other types of uncertainty invoked different responses: uncertainty arising from ambiguity—for example, varied recommendations—and complexity—the vagueness of clinical descriptors, or the difficulty in judging patient understanding. Many GPs preferred to negotiate or transfer responsibility for these uncertainties. For example, a GP experiencing uncertainty in a testing decision might tell the patient about discordant recommendations to justify their uncertainty and immediately refer to specialists for further opinion. Some GPs appeared to hold that responsibility for ambiguity or complexity should be collective and others should be involved in negotiating decisions, or they preferred to transfer those decisions to specialists. Ambiguity and complexity commonly led to practical issues for GPs arising from this uncertainty–how to engage in ‘good’ practice and appropriate communication based on complex evidence and ambiguous guidance. Ambiguity and complexity were also a foundation for GPs’ sense of personal burden, as some GPs expressed anxiety about their capacity to make ‘good’ decisions without good evidence, and ability to judge what level of information was suitable to support consent. Theoretically these sources of uncertainty could be modified or improved with system change (for example, consistent recommendations, established consent protocols) but GPs felt limited in their capacity to make changes at the clinical level.

## Discussion

GPs described experiencing considerable and, at times, burdensome uncertainty in the context of PSA testing of asymptomatic men for prostate cancer. Locating and describing sources or types of uncertainty (Table 1) as per Han is important in this context because the various types of uncertainty produced different issues (Table 2), which GPs responded to and managed using distinct strategies (our 3 strategies outlined above).

### Sources, Issues, and Management of Uncertainty

GPs in our study appeared to manage uncertainty arising from probabilistic information with reasonable confidence when compared to the other sources of uncertainty. One possible explanation for this finding is that GPs are familiar with probabilities and are trained to interpret and manage probabilistic information in the clinical setting. Numerous resources are available presenting probabilistic and statistical information in multiple formats (numerically, graphically) to assist GPs and to support the decision making capacity of their patients. While probabilistic uncertainty is particularly challenging in relation to PSA testing and cannot be readily resolved with leaflets or information sheets, we propose that such resources ‘normalise’ the probabilistic uncertainty inherent to this context. It is possible that GPs feel more comfortable taking charge or engaging with others about probabilistic uncertainty because they are better able to get a handle on the uncertainty they are dealing with because it is an ongoing and familiar type of uncertainty. Probabilistic information about outcomes is also the typical kind of information assumed to be shared in processes of shared decision-making.

It is clear from our results that uncertainty from ambiguity on the other hand was extremely frustrating for GPs; GPs were uncertain about guidance from medical authorities and were unclear of their clinical and legal obligations. These uncertainties are grounded in ambiguity, which, as the decision psychology literature has shown, people generally prefer to avoid (e.g. [25]. Ambiguity is more directly related to uncertainties about what to do in practice than probabilistic uncertainty, and perhaps leaves more scope for GPs to take a “wrong” course of action. Aspects of ambiguity in this context are potentially modifiable, which could reduce some uncertainty for GPs. For example, clearer and more consistent expert guidance may assist. Yet even if more ‘certainty’ was implemented at the system-level via clear guidelines or consistent advice from authoritative sources, clinicians will inevitably still experience some personal uncertainty stemming from the complexity of this information, and because of diverse GP and patient value systems in the clinical context (e.g. questioning appropriateness of recommendations).

Complexity was also experienced as particularly uncomfortable for GPs; some GPs described feeling uncertain about their own clinical judgment. Complexity is essentially a source of uncertainty amenable to individual value judgment, interpretation, and assumptions. It is therefore difficult to offer training or advice to GPs about ideal management of complexity uncertainty in relation to PSA testing. While clearer expert opinion may lessen ambiguity, GPs are left to deal with complexity at the clinical level with the individual patient. Our data from UK GPs illustrates that even with system-level guidance and clear ‘best practice’ formulations, GPs continue to experience personal uncertainty because every patient and consultation involving PSA testing is different and involves difficult value judgments. Clinicians will invariably face ongoing uncertainty about the nature of medical evidence and individual and distributive health care [26].

### The Burden of Uncertainty

Ordering PSA tests for screening purposes is paradoxical in relation to uncertainty. Testing is a response to uncertainty: an attempt to better predict a man’s risk of developing prostate cancer. Yet while PSA testing aims to reduce uncertainty, the characteristics of the test when used as a screening tool mean that it tends to introduce more scientific, practical, and personal uncertainty than it eliminates. Faced with a PSA test result, the clinical significance of which is intrinsically uncertain, some GPs actively tried to lessen uncertainty by looking to guidelines on which to base decisions, or by sharing their knowledge with patients. However these strategies also tended to compound uncertainty, piling up knowledge about the uncertainties regarding outcomes, and GPs felt further burdened as a result. This is the great paradox of the PSA. If GPs chase certainty via more and more testing and investigation, this may create more uncertainty. Testing can undoubtedly create certainty for some GPs and patients at the individual level, but it comes at a cost of lots of uncertainty, including GPs’ top line uncertainty about using the PSA test at all. This, as our research has demonstrated, is what inflicts considerable burden on GPs: because they have burdened patients with information which cannot provide any clear answer, because their efforts to locate certainty have gone unrewarded, and because of remaining uncertainty about what counts as good practice.

Uncertainty and the associated struggle is not just a burden to be borne by individual practitioners. There is an expectation that GPs will practice in line with professional standards and evidence-based medicine. If GPs are expected to act according to these standards, it would be reasonable to suppose that the health care system might owe GPs some reciprocal supports to make it possible for them to do so.

### Ethical Considerations

There are judgments to be made about how best to involve men in PSA testing decisions. Our data touches on uncertainties about whether, when, and in what forms communicating about uncertainty is considered appropriate. Communicating uncertainty is not simply about probabilities; ambiguity and complexity are also key sources of GP uncertainty and subsequent burden. Any attempt to guide communication about uncertainty in PSA testing practice may be more effective if it addresses all three sources of uncertainty.

Ethical questions remain in relation to presenting uncertainty to patients who wish to avoid making medical decisions; coping with uncertainty is known to be a source of substantial stress and anxiety for many patients (e.g. [27]. Manson and O’Neill argue that a trusting and responsive relationship might be more valued by patients than detailed information exchange [28]. Perhaps in some situations, a “taking charge” approach, whereby GPs call on their professional experience and apply ‘rules of thumb’ to direct their practice might be appropriate, while in more complex situations, GPs may need to discuss and reach agreement with others regarding how decisions based on uncertainty will be allocated and resolved.

More work is needed to clarify under what circumstances uncertainty should be communicated to patients, and if so what aspect of the uncertainty construct should be addressed. Parascandola et al argue that respectful interaction with patients requires disclosure about uncertainty even when it is not offered to gain consent or in the service of patient decision making; as long as patients understand the general decision making context [29]. Ideally, doctors need to feel supported in their dealings with uncertainty. Research suggests that when doctors are comfortable with uncertainty and collaborate with patients in their medical care, patient trust and satisfaction are high [29].

### Limitations

This analysis was inductive rather than commencing with research questions about uncertainty. Further research could test our findings and explore the impacts of uncertainty in more depth.

## Conclusions

These important aspects of uncertainty require further and specific investigation, including the potential implications of clinician uncertainty for cost and quality of health care [30].

This study is a first step in mapping how clinicians practice under conditions of uncertainty. Our unique findings identified what doctors actually do in response to the different types of uncertainty encountered. These results have practical value: the GPs in our study responded to the various types of uncertainty and their associated issues differently. It seems likely that GPs and their patients will benefit from greater acknowledgment by the profession of specific sources of uncertainty and their unique implications, and in particular the often-neglected uncertainty arising from ambiguity or complexity. Achieving this would be an essential step in promoting GP engagement with uncertainty, and ultimately patient involvement in better informed decisions about PSA testing for prostate cancer.
